# X-linked adrenoleukodystrophy and primary adrenal insufficiency

**DOI:** 10.3389/fendo.2023.1309053

**Published:** 2023-11-16

**Authors:** Marco Cappa, Tommaso Todisco, Carla Bizzarri

**Affiliations:** ^1^ Research Area for Innovative Therapies in Endocrinopathies, Bambino Gesù Children’s Hospital, IRCCS, Rome, Italy; ^2^ Unit of Paediatric Endocrinology, Bambino Gesù Children’s Hospital, IRCCS, Rome, Italy

**Keywords:** X-linked adrenoleukodystrophy, primary adrenal insufficiency, very long chain fatty acids, adrenal function, cortisol replacement

## Abstract

X-linked adrenoleukodystrophy (X-ALD; OMIM:300100) is a progressive neurodegenerative disorder caused by a congenital defect in the ATP-binding cassette transporters sub-family D member 1 gene (ABCD1) producing adrenoleukodystrophy protein (ALDP). According to population studies, X-ALD has an estimated birth prevalence of 1 in 17.000 subjects (considering both hemizygous males and heterozygous females), and there is no evidence that this prevalence varies among regions or ethnic groups. ALDP deficiency results in a defective peroxisomal β-oxidation of very long chain fatty acids (VLCFA). As a consequence of this metabolic abnormality, VLCFAs accumulate in nervous system (brain white matter and spinal cord), testis and adrenal cortex. All X-ALD affected patients carry a mutation on the ABCD1 gene. Nevertheless, patients with a defect on the ABCD1 gene can have a dramatic difference in the clinical presentation of the disease. In fact, X-ALD can vary from the most severe cerebral paediatric form (CerALD), to adult adrenomyeloneuropathy (AMN), Addison-only and asymptomatic forms. Primary adrenal insufficiency (PAI) is one of the main features of X-ALD, with a prevalence of 70% in ALD/AMN patients and 5% in female carriers. The pathogenesis of X-ALD related PAI is still unclear, even if a few published data suggests a defective adrenal response to ACTH, related to VLCFA accumulation with progressive disruption of adrenal cell membrane function and ACTH receptor activity. The reason why PAI develops only in a proportion of ALD/AMN patients remains incompletely understood. A growing consensus supports VLCFA assessment in all male children presenting with PAI, as early diagnosis and start of therapy may be essential for X-ALD patients. Children and adults with PAI require individualized glucocorticoid replacement therapy, while mineralocorticoid therapy is needed only in a few cases after consideration of hormonal and electrolytes status. Novel approaches, such as prolonged release glucocorticoids, offer potential benefit in optimizing hormonal replacement for X-ALD-related PAI. Although the association between PAI and X-ALD has been observed in clinical practice, the underlying mechanisms remain poorly understood. This paper aims to explore the multifaceted relationship between PAI and X-ALD, shedding light on shared pathophysiology, clinical manifestations, and potential therapeutic interventions.

## 1 Introduction

### 1.1 X-linked adrenoleukodystrophy

X-linked adrenoleukodystrophy (X-ALD; OMIM:300100) is a progressive neurodegenerative disorder caused by a congenital defect in the ATP-binding cassette transporters sub-family D member 1 gene (ABCD1) producing adrenoleukodystrophy protein (ALDP). According to population studies X-ALD has an estimated birth prevalence of 1 in 17.000 subjects (considering both hemizygous males and heterozygous females), and there is no evidence that this prevalence varies among regions or ethnic groups (1). ALDP deficiency results in a defective peroxisomal β-oxidation of very long chain fatty acids (VLCFA). As a consequence of this metabolic abnormality, VLCFAs accumulate in different critical parts of the body, such as CNS (brain white matter and spinal cord), testis and adrenal cortex. All X-ALD affected patients carry a mutation on the ABCD1 gene. Nevertheless, patients with a defect on the ABCD1 gene can have a dramatic difference in the clinical presentation of the disease. In fact, X-ALD can vary from the most severe cerebral pediatric form (CerALD), to adult adrenomyeloneuropathy (AMN), Addison-only and asymptomatic forms.

### 1.2 Primary adrenal insufficiency

Primary adrenal insufficiency (PAI), also known as Addison’s disease, is a rare and chronic endocrine condition characterized by the inadequate adrenal production of steroid hormones, due to damage or dysfunction of the adrenal gland. The prevalence of PAI varies among different countries, with the greatest prevalence in countries of northern Europe, where a prevalence of 15-20 affected individuals per 100.000 has been described (2). The most common cause of acquired PAI is autoimmune destruction of the adrenal gland, mediated by antibodies targeting the 21-hydroxylase enzyme (3). This condition can occur at any age, with most individuals affected between the age of 20 and 50 (4). Along with the acquired causes, PAI can be the result of inherited disorders as well. The most common form of inherited PAI is congenital adrenal hyperplasia, which refers to a heterogenous group of genetic conditions characterized by defects in different enzymes involved in adrenal and gonadal steroidogenesis. X-ALD represents a relevant cause of PAI in male children and adults (5–8). The proportion of cases in which PAI is related to ALD is age-dependent. ALD is the most common cause of adrenal insufficiency in boys before 15 years of age [18]. Adrenal function is abnormal in 90% of neurologically symptomatic ALD boys and in 70% of men with AMN. It is usually normal in female carriers (9, 10).

### 1.3 Objective of the paper

PAI and X-ALD are two distinct yet interconnected medical conditions. Although the association between these two disorders has been observed in clinical practice, the underlying mechanisms and the extent of their relationship remain poorly understood. This paper aims to explore the multifaceted relationship between PAI and X-ALD, shedding light on their shared pathophysiology, clinical manifestations, and potential therapeutic interventions. By addressing several key objectives, this review aims to contribute to the knowledge and understanding of these complex disorders, ultimately improving patient care and outcomes.

## 2 Pathophysiology of X-linked adrenoleukodystrophy

### 2.1 Genetic basis and inheritance pattern of X-ALD

X-ALD is primarily caused by mutations in the ABCD1 gene located on the X chromosome (Xq28), which spans for 19.9 kb and 10 exons (11). The mutations in the ABCD1 gene exhibit a wide spectrum, including missense, nonsense, frameshift, and splice-site variants. Type and location of the variant affect the severity and phenotypic variability of X-ALD (12). Nonetheless, identical variants can result in highly diverse clinical phenotypes, suggesting the presence of additional factors that influence the expression of the disease (13). The majority of affected individuals have mutations resulting in loss of ALDP protein function, while a smaller proportion may have milder mutations allowing some residual protein activity. X-ALD follows an X-linked inheritance pattern due to the location of the ABCD1 gene on the X chromosome. Nevertheless, some patients are affected by a *de novo* mutation, indicating that the mutation can occur in the germ line. Since X-ALD is an X-linked inherited disorder, males are more severely affected than females. Some heterozygous X-ALD females can exhibit symptoms due to skewed X-chromosome inactivation or other genetic factors.

### 2.2 Impact of VLCFA accumulation on adrenal function

ALDP is a constitutive part of the peroxisomal membrane. It transports VLCFAs from the membrane to the matrix where they eventually undergo β-oxidation. If this mechanism is hampered, the breakdown of VLCFAs is impaired, resulting in a rise of cytosolic VLCFAs concentrations. The precise role of the accumulation of VLCFAs in the pathogenesis of ALD remains unclear. Both a direct and indirect effect on cells survival and functioning has been postulated. A direct cytotoxic effect on oligodendrocyte and astrocytes has been observed in rats, following exposure to C26:0 lipids (14). Similarly, supraphysiological accumulation of C26:0 in astrocytes of ABCD1-deficient mice is followed by production of high reactive oxygen species (15, 16). These findings suggest a pivotal role of VLCFAs accumulation in the pathogenesis of ALD. It is still unknown why only few specific sites of the body are affected by the disease, while ABCD1 gene is widely expressed and VLCFAs are elevated in several other tissues (17).

The exact mechanism whereby increased levels of VLCFAs lead to toxicity in the adrenal glands is not well understood. However, it has been observed that cholesterol, along with saturated VLCFAs, accumulates in the zona fasciculata and reticularis of the adrenal glands. This accumulation starts during fetal development (18). VLCFAs preferentially accumulate in the postnatal zona fasciculata and zona reticularis, which are responsible for the production of glucocorticoids (cortisol) and androgens, respectively. Over time, this chronic accumulation is believed to trigger apoptosis and eventual shrinkage of the adrenal cortex, leading to a decrease in cortisol production (19). This observation provides further evidence supporting the idea that the buildup of VLCFAs plays a significant role in the development of adrenal insufficiency. Furthermore, function of adrenal cells has been witnessed to be disrupted by altered microviscosity of the cell-membrane when exposed to C26:0, leading to an impaired response to adrenocorticotropin (ACTH) stimulation (20). Another proposed mechanism for glucocorticoid and androgen deficiencies is a relative lack of cholesterol necessary for their production, as cholesterol is a degradation product of cholesterol esters containing VLCFA (21).

## 3 Clinical manifestations and diagnosis of X-ALD

### 3.1 Neurological manifestations of X-ALD

X-ALD primarily affects the white matter of the CNS, leading to neuroinflammation, demyelination and subsequent neurological symptoms. In approximately 85% of the individuals with clinical symptoms, brain magnetic resonance imaging (MRI) shows a characteristic pattern of symmetric confluent hyperintense lesions in T2 and FLAIR images, usually first appearing in the parieto-occipital region of both hemispheres. Contrast enhancement.is evident at the edges of the lesions, indicating inflammation and disruption of the blood-brain barrier. The progression of these lesions is unpredictable; it can be severe and rapid in the childhood cerebral form; milder and slower in forms with adult onset (11, 22).

The manifestations of X-ALD can be broadly categorized into different clinical phenotypes:

#### 3.1.1 Cerebral ALD (CerALD)

This is the most severe form of X-ALD, typically affecting young boys (typically between 4 and 8 years of age). It is characterized by progressive inflammatory demyelination in the cerebral white matter. Symptoms initially include behavioural changes, learning difficulties, and attention deficits. As the disease progresses, individuals may develop motor abnormalities, impaired vision, hearing loss, seizures, and cognitive decline. In advanced stages, CerALD can lead to severe disability and loss of motor function (11, 22).

#### 3.1.2 Adolescent and Adult Onset X-ALD

Adolescent and adult onset X-ALD occurs later in life and has a milder and slower progression compared to CerALD. Neurological symptoms include behavioural changes, impaired coordination, gait abnormalities, and cognitive decline. Individuals with this form of X-ALD may also experience sensory abnormalities, such as loss of vibration and positional sense. Although these forms have been classified as separated forms, nowadays many authors tend to consider them as variants of CerALD (adolescent onset X-ALD) and of AMN (adult onset X-ALD). (11, 22)

#### 3.1.3 Adrenomyeloneuropathy (AMN)

AMN is the most common form of adult-onset X-ALD. It primarily affects hemizygous males, although heterozygous females can also be affected to a lesser extent, with a similar but later progression of symptoms (usually after the 4^th-^or 5^th^ decade of life). It is characterized by spinal cord involvement, leading to progressive motor dysfunction and sensory deficits. Symptoms typically include weakness, muscle stiffness, difficulty walking, bladder dysfunction, and sexual dysfunction (11, 22). Patients with AMN may also present primary hypogonadism, characterized by progressive impairment of Leydig cell function, decreased testosterone levels and increased LH and FSH levels. Fertility has been described as normal in the pre-symptomatic period and gradually impaired with the progression of the disease. In men with AMN and late onset of the disease, the possibility of procreation and the resulting inheritance by their daughters should be considered. All families should have genetic counseling regarding the inheritance of X-ALD (23). Cognitive impairment is generally absent at presentation, but a secondary cerebral involvement may appear during the progression of the disease (24).

### 3.2 Adrenal involvement in X-ALD

The endocrine complications of X-ALD extend beyond its neurological impact, encompassing adrenal dysfunction and hypogonadism. These complications significantly contribute to the disease burden and require diligent monitoring and multidisciplinary management. Early identification, appropriate hormone replacement therapy, and continuous medical oversight are crucial to mitigate the impact of these complications and enhance the quality of life for X-ALD patients. In X-ALD, PAI is a dominant clinical aspect. During an assessment of adrenal function in a group of 49 affected boys who had not yet exhibited neurological symptoms (ranging in age from 5 months to 13 years), it was observed that 80% of them already displayed biochemical evidence of PAI, which otherwise did not manifest as clinical symptoms, at the point of their ALD diagnosis (25). Due to the absence of comprehensive prospective investigations into the natural progression of the disease, the extent to which PAI develops in individuals with X-ALD remains uncertain, with different research studies indicating a penetrance of 50-100% (26). In particular, it has been observed that the possibility of X-ALD patients to develop PAI fluctuates across different stages of life, with a higher risk occurring during the initial 10 years. According to an international retrospective review of medical records of affected boys and male adults, the cumulative probability of experiencing PAI reached its peak by the age of 10 years (46.8%), remained notably elevated up to 40 years of age (an additional 28.6%), and subsequently declined significantly beyond that point (an additional 5.6%) (26). While plasma levels and ratios of VLCFAs are indicative of X-ALD, they appear not to correlate with the risk of developing AI, spinal cord disease, or cerebral disease based on age, as demonstrated by different research groups (27, 28). PAI was identified in X-ALD patients as young as 7 months and 5 months old. These individuals showed biochemical irregularities associated with PAI but yet did not exhibit any clinical symptoms (25, 26). Although studies on female X-ALD patients are few in number, PAI remains an exceedingly uncommon condition in heterozygous females (29–31). In our cohort of 49 female carriers we observed only 2 patients with PAI. While PAI is commonly associated with deficits in both glucocorticoid and mineralocorticoid functions, it’s worth noting that in cases of X-ALD related PAI, the mineralocorticoid function can remain unaffected (9, 10). As VLCFAs mainly accumulate in the zona fasciculate and reticularis, the relative preservation of the zona glomerulosa aligns with the observation that mineralocorticoid function remains functional in approximately 50% of the patients (26). It is noteworthy that the category formerly referred to as “Addison-only” is now regarded as rare. By definition, these patients are devoid of detectable neurological involvement. Nevertheless, due to the progressive nature of the disease, a significant number of individuals within this category eventually experience neurological manifestations (32).

### 3.3 Diagnostic approaches for adrenal insufficiency and X-ALD/AMN

The concurrent presence of PAI, neurological symptoms and characteristic signs at brain MRI serves as a diagnostic indicator that typically leads to X-ALD diagnosis. However, the complex variability in the clinical presentation means that diagnosis across different age groups may rely on varying clinical features. In children and adults, cognitive and neurological symptoms that could potentially indicate CerALD include the sudden emergence of attention and learning problems, the onset of behavioural issues, deteriorating speech and vision and progressive difficulties with walking and coordination. Of note, PAI as well can manifest with neurological overlapping symptoms, such as malaise, fatigue and impairment in cognitive function up to confusion. A distinguishable clinical characteristic of PAI is the increased pigmentation of the skin and mucous membranes, particularly in regions exposed to sunlight and friction due to the elevated levels of circulating ACTH, that can be the first clinical manifestation of X-ALD. When suspected, the diagnosis of PAI follows the same diagnostic guidelines used for other causes of PAI. As aforementioned, dosing plasma renin and aldosterone remains important to evaluate functioning of the zona glomerulosa. In the presence of indicative signs or symptoms of X-ALD, the diagnosis can be confirmed through biochemical and genetic assessment. Elevated levels of VLCFAs in boys and men lead to unequivocal diagnosis. VLCFA levels and ratios are in the normal range in 10-15% of female carriers (33).

ABCD1 genetic testing is recommended to confirm the diagnosis. A detection of a known pathogenic ABCD1 variant validates the diagnosis. Nevertheless, *de novo* mutations and variants of uncertain significance (VUS) are common. In these cases, the diagnosis can be made when the putative causative mutation is associated with typical symptoms. Thus, diagnosis can be difficult in asymptomatic females with either VUS or *de novo* mutations. In these patients, *in vitro* fibroblasts studies can help studying the pathogenicity of the putative variant.

In cases where PAI is diagnosed in the absence of neurological symptoms, the question of who should be tested for VLFCA levels still remains a subject of debate. Among children with PAI of unknown origin, 2 out of 47 boys were found to have ABCD1 mutations consistent with X-ALD with no indicative neurological symptoms (34). This discovery suggests the importance of incorporating VLCFA assessment for male children diagnosed with PAI. Nevertheless, indications about testing children with PAI vary according to different societies and research groups. The Endocrine society guidelines for PAI (35) suggest dosing VLCFA in males with confirmed PAI and negative 21-OH antibodies (21OHab) when older than 6 months. According to the same source, VLCFAs measurement should be present in the first evaluation in the case of preadolescent boys. A recent influential Seminar (36) suggests dosing VLCFAs in male patients with PAI negative for 21OHab and with normal computed tomography scan negative for adrenal enlargement. A recent international consensus (37) recommends testing all male children and adults with PAI and negative 21-hydroxylase autoantibodies or other organ specific autoantibodies. The same paper suggests not to routinely test female patients with PAI, as PAI is considered rare in heterozygous females.

According to the same consensus, in the case of known X-ALD without detected PAI, periodic screening should be started by the age of 6 months in males, with combined evaluation of basal glucocorticoid and mineralocorticoid function every 3 to 6 months until the age of 10 years. From pubertal age to adulthood, patients should be tested yearly. Of note, no routine screening is recommended in female patients. According to some authors of the aforementioned consensus, routine screening should be performed until the age of 40 years and solely if supported by symptoms after age 41 (26). Notably, Capalbo et al. reported that 32.1% of the patients were diagnosed with PAI by stimulation testing (8).

## 4 Management and treatment of X-linked adrenoleukodystrophy-associated adrenal insufficiency

### 4.1 Glucocorticoid replacement

When PAI is confirmed, hormone replacement therapy is recommended in order to prevent serious life-threatening events. As hormone replacement therapy in X-ALD does not vary significantly from PAI caused by other conditions, it is mandatory to start hormone treatment even without an established diagnosis of X-ALD. As for glucocorticoid deficiency, treatment varies in different age groups, according to the most recent guidelines. In children, glucocorticoid replacement therapy (GCCrt) should be started with hydrocortisone (HC) at a total daily dose of 10 mg/m^2^, with dose titration according to individual needs (35). The normalization of ACTH levels should not be pursued, as for the relative resistance of the adrenal gland to ACTH, the normalization would be achieved only at the cost of a significant HC overdose. Synthetic or long-acting glucocorticoids should not be used for GCCrt in children, because of their negative impact on linear growth and puberty. It is worth noting that during puberty daily requirement of HC can vary as a consequence of endogenous sexual steroids production, so that dose titration may be necessary (38). Treatment effectiveness in children with PAI must be carefully monitored by clinical evaluation of growth velocity, weight, blood pressure and energy status every 3 to 6 months (39). Notably, in children with CerALD growth and general wellbeing can be hampered by the condition itself, hence a cautious and reasonable approach may be routinely check electrolytes and carbohydrate balance, in order to detect signs of undertreatment in children with ambiguous symptoms or signs. In adults with PAI and X-ALD, GCCrt doses vary according to the treatment of choice. Standard regimens consist of oral HC (15–25 mg/day) or cortisone acetate (20–35 mg/day) in two or three divided doses. Treatment adequacy should be monitored yearly in adults with PAI. Since adult X-ALD patients can suffer for hypogonadism, which can be associated with fatigue and low energy levels, routine follow up should involve gonadal function, electrolyte balance and glucose metabolism, in order to discriminate symptoms in complex patients.

### 4.2 Mineralocorticoid replacement

As the adrenal zona glomerulosa can be spared from disruption, plasma renin and aldosterone levels and serum electrolyte should be always checked before considering the initiation of mineralocorticoid replacement therapy (37). When started, therapy relies on fludrocortisone (FC) oral therapy, at a starting dose of 100 mcg in children and 50-100 mcg in adults, administered once daily in the morning (40). As PAI in children with X-ALD can occur early in life, oral supplement of sodium chloride 1gr/day should be added in infants up to 1 year of age.

Given the disruption of the zona reticularis, a putative role for the supplementation of adrenal sex steroids may be hypothesized. As far as we are aware of, there is neither clear evidence nor strong recommendation for the use of dehydroepiandrosterone (DHEA) in PAI.

### 4.3 Emerging therapies and potential future treatments

HC therapy has been the mainstay of treatment of PAI in X-ALD. However, novel approaches have been explored to fine-tune hormonal replacement and mimic the body’s natural rhythm more closely and to ameliorate patient’s compliance and wellbeing. This includes the development of glucocorticoid formulations providing a more physiological hormone release, thus minimizing side effects and optimizing therapeutic benefits. The tablets commercially available in the market come with a minimum dosage of 5 mg in the USA and Europe. This dosage disparity presents a challenge when it comes to achieving precise dosing for infants and young children, as it limits the ability to finely adjust the dosage according to their specific requirements. A granulated HC formulation has recently gained approval in Europe. This formulation offers small dosages of 0.5, 1, 2, and 5 mg, which can prove useful in children taking small quantities of HC (41).

Adults with PAI for X-ALD can benefit from the dual-release HC formulation (DRHC), consisting in a system based on an outer layer for immediate release and an inner retard formulation. DRHC has proved in comparative studies to have a more natural cortisol profile. Hitherto, this formulation is considered off-label in children with PAI (42).

Lorenzo’s oil, that is a mixture of monounsaturated erucic acid (C22:1) in the form of triglycerides (glyceroltrierucate), in conjunction with a mixture of monounsaturated oleic acid (C18:1) also in triglyceride structure (glyceroltrioleate), at a 4:1 ratio, was shown to normalize plasma C26:0 levels within one month when accompanied by a low-VLCFA diet (43). However, the effectiveness of this therapeutic approach has been met with skepticism, as Lorenzo’s oil fails to halt the progression of pre-existing neurological symptoms, so that hitherto it is not routinely recommended by X-ALD guidelines (37). The potential benefit of Lorenzo’s oil in regard to PAI has not been deeply investigated, and definitive evidence is still lacking. Nevertheless, in a perspective study on a small group of adult males with AMN, Lorenzo’s oil supplementation was able to lower ACTH levels in patients with subclinical PAI, potentially linking VLCFA levels to the degree of adrenal dysfunction (44). X-ALD related PAI is probably due to a defective adrenal response to ACTH, related to VLCFA accumulation with progressive disruption of the adrenal cell membrane functions. Lorenzo’s oil therapy may be able to improve VLCFA clearance and restore ACTH receptor activity in an early phase. Recently, a new nutritional approach has been proposed with a new oil mixture allowing erucic acid to cross the blood brain barrier and reduce VLCFA levels in spinal fluid (45). No data on the effect of this nutritional approach on adrenal function has been published.

Allogenic hematopoietic cell transplantation (HCT) has the potential to arrest the progression of the disease in individuals with inflammatory cerebral involvement. The post-HCT consequences are intrinsically linked to the neurological condition during the transplantation phase. Patients displaying mild neurological impairment along with discernible MRI involvement appear to achieve the most favorable outcomes (46, 47). Gene therapy is a cutting-edge approach that has shown potential in various genetic disorders, including X-ALD. A lentiviral vector has been used to introduce a wild-type copy of the ABCD1 gene into the patients’ hematopoietic stem cells *ex vivo*. Subsequently, the genetically modified cells have been reintroduced into the patients (48).

We still do not fully understand how patients with cerebral ALD may achieve disease stabilization after HCT or gene therapy. Improvement in disease burden is usually not observed, and stop or minimization of progression represent the primary therapeutic goals (49). There is little data to support that myelin producing oligodendrocytes of the CNS are successfully replaced through allogeneic HCT. Patients that have undergone transplantation and fail to engraft with donor-derived cells experience progression of neuroinflammation and white matter damage, suggesting that the immune suppression associated with allogeneic HCT may decrease neuroinflammation. On this basis, the presence of a cell product expressing the wild-type ABCD1 appears critical in achieving stabilization of the disease. The ABCD1 gene product (ALD protein, ALDP) is incorporated in the peroxisomal membrane and is not released into the environment with the ability to provide ‘cross correction’ of adjacent cells. It remains to be elucidated whether cells expressing wild-type ABCD1 are able to localize to the brain and specifically reduce VLCFAs in the CNS, or they simply play a role in stabilizing inflammation, enhancing cellular respiration, and reducing oxidative stress (49).

In the context of PAI in X-ALD, HCT and gene therapy may potentially restore the impaired adrenal function by delivering functional genes to the adrenal glands. There are no long-term observational studies after HCT and there is no evidence so far that HCT can change the course of PAI in ALD patients. All HCT-treated patients with pre-existing PAI had to continue GCCrt after transplantation. No cases of reversed PAI have been reported in X-ALD patients who underwent gene therapy.

As already said, the mechanisms whereby elevated VLCFAs damage the steroid producing cells of the adrenal glands and testicles are not fully understood. It has been demonstrated that the accumulation of cholesterol and saturated VLCFAs in the zona fasciculata and reticularis of the adrenal cortex starts during foetal life (18), probably in conjunction with the activation of foetal adrenal steroidogenesis. This chronic accumulation triggers an early apoptosis of the adrenal cortical cells leading to irreversible shrinkage of the adrenal cortex (19). A similar (even if later) process may be at the basis of the chronic Leydig cell damage leading to adult-onset hypogonadism in patients with AMN.

While this field is still in its infancy and faces several challenges, ongoing research and advancements offer a glimpse into a future where gene therapy could dramatically change PAI outcome.

### 4.4 Newborn screening

HCT is potentially life-saving in patients with ALD, if initiated as soon as cerebral disease is discovered. However, as neurological impairment does not improve after HCT, an early diagnosis would allow for surveillance of cerebral disease and reduce potential residue (46). Neonatal screening for ALD was initially proposed by Moser and colleagues in 2004-2005 (50). However, at that time there was no valid test for ALD using the newborn blood spot. In the following years, a screening test for ALD by measurement of C26:0-lysophosphatidylcholine, C26:0-LPC in newborn dried blood spot was established (51). After refining the liquid chromatography-tandem mass spectrometry (LC-MS/MS) assay of C26:0-LPC and several pilot studies, in December 2013, neonatal screening for ALD was started in the state of New York. From that time onward, some countries have implemented their neonatal screening program with ALD screening (52).

The early detection of the characteristic biochemical abnormalities associated with ALD and AMN has proven to be reliable to detect affected subjects but also poses new clinical ethical issues (52). Considering the variable and unpredictable clinical expression and prognosis of the disease, the justification of neonatal screening could be questionable and it is still a matter of discussion.

## 5 Conclusion

X-ALD is a complex progressive genetic disorder with a wide spectrum of clinical phenotypes.

While the neurological deterioration is a dominant clinical aspect of X-ALD, PAI and endocrine complications contribute significantly to the disease burden. The development of PAI in X-ALD can occur early, even in the absence of clinical symptoms, highlighting the importance of early screening and monitoring. Diagnosis of X-ALD relies on the assessment of VLCFA levels, genetic testing, and clinical presentation. Hormone replacement therapy remains the cornerstone of treatment for PAI in X-ALD. The dosing and management of glucocorticoid and mineralocorticoid replacement therapies differ based on age groups and individual needs. Emerging therapeutic approaches, such as modified-release formulations and gene therapy, hold promise for improving treatment outcomes and addressing the complexities of PAI in X-ALD.

While advancements have been made in understanding and managing X-ALD, further research is essential to unravel the intricate mechanisms underlying VLCFA toxicity and adrenal dysfunction. The interplay of genetics, hormone pathways, and disease progression requires continued exploration to develop more targeted and effective therapeutic strategies. As we navigate the intricate landscape of X-ALD, a multidisciplinary approach, encompassing medical, genetic, and endocrinological expertise, will be pivotal in improving the lives of individuals affected by this complex disorder.

## Author contributions

MC: Validation, Writing – review & editing. TT: Writing – original draft. CB: Conceptualization, Supervision, Writing – review & editing.
